# Effect of low‐dose terazosin on arterial stiffness improvement: A pilot study

**DOI:** 10.1111/jcmm.18547

**Published:** 2024-07-23

**Authors:** Yuqi Guan, Yucong Zhang, Liangkai Chen, Yazhi Ren, Hao Nie, Tianyi Ji, Jinhua Yan, Cuntai Zhang, Lei Ruan

**Affiliations:** ^1^ Department of Geriatrics, Tongji Hospital, Tongji Medical College Huazhong University of Science and Technology Wuhan China; ^2^ Key Laboratory of Vascular Aging, Ministry of Education, Tongji Hospital, Tongji Medical College Huazhong University of Science and Technology Wuhan China; ^3^ Department of Nutrition and Food Hygiene, Hubei Key Laboratory of Food Nutrition and Safety, School of Public Health, Tongji Medical College Huazhong University of Science and Technology Wuhan China; ^4^ Ministry of Education Key Lab of Environment and Health, School of Public Health, Tongji Medical College Huazhong University of Science and Technology Wuhan China

**Keywords:** arterial stiffness, brachial‐ankle pulse wave velocity (BaPWV), drug effectiveness analysis, pilot study, vascular ageing

## Abstract

Arterial stiffness, a prominent hallmark of ageing arteries, is a predictor of all‐cause mortality. Strategies for promoting healthy vascular ageing are encouraged. Here we conducted a pilot study to evaluate the potential effects of low‐dose Terazosin on arterial stiffness. We enrolled patients aged over 40 with elevated arterial stiffness, defined as a brachial‐ankle pulse wave velocity (baPWV) ≥1400 cm/s, who were administered Terazosin (0.5 and 1.0 mg/day) from December 2020 to June 2023. Treatment responses were assessed every 3 months. Linear regression analysis was used to characterise the improvement. We matched cases who took Terazosin for 1 year with Terazosin‐free controls using propensity score matching (PSM). Our findings demonstrate that Terazosin administration significantly affected arterial stiffness. (1) Arterial stiffness significantly improved (at least a 5% reduction in baPWV) in 50.0% of patients at 3 months, 48.6% at 6 months, 59.3% at 9 months, and 54.4% at 12 months, respectively. (2) Those with higher baseline baPWV and hypertension exhibited a significantly reduced risk of non‐response. (3) Terazosin was associated with a reduction of baPWV at 1‐year follow‐up (linear regression: *β* = −165.16, *p* < 0.001). This pilot study offers valuable insights into the potential significance of Terazosin in improving arterial stiffness and paves the way for future randomised clinical trials in combating vascular ageing.

## 1 INTRODUCTION

The ageing population poses a formidable challenge to global health.^1^ The ageing of the vascular system results in structural and functional remodelling, primarily characterised by arterial stiffness. Vascular stiffness and vascular function alter in association with ageing, accompanied by a gradual deterioration of its protective mechanisms.^2^ The stiffening of the vessel wall impairs its cushioning function, leading to the progression of chronic cardiovascular diseases.^3^, ^4^ BaPWV, a non‐invasive measure of arterial stiffness, stands out as a robust and independent predictor of cardiovascular morbidity and mortality, surpassing traditional risk factors.^5^, ^6^

Recent advancements have generated increased interest in promoting healthy vascular ageing through ameliorating vascular stiffness.^7^ Current therapeutic approaches primarily focus on managing associated risk factors of vascular ageing or stiffness. For instance, statins and antihypertensive agents appear to reduce arterial stiffness in hypertensive patients.^8^, ^9^, ^10^ However, the efficacy of these interventions in addressing vascular stiffness remains controversial and inadequate.^11^

Several cellular and functional changes contribute to the ageing process of vasculature.^12^ Maintaining the proper function and structure of the vascular system relies on vascular smooth muscle cells^13^ and endothelial cells.^14^ These cells regulate membrane transport and barrier function through their proper metabolic processes, particularly energy metabolism.^15^ Terazosin, an α 1‐adrenergic receptor antagonist, has demonstrated efficacy in augmenting glycolysis by activating an initial ATP‐producing enzyme in the glycolytic pathway, thereby increasing intracellular ATP levels,^16^ and exhibiting potential therapeutic effects in diseases such as Parkinson.^17^, ^18^, ^19^ Increasing ATP could play a role in retarding cellular ageing,^20^ as well as enhancing the activity of endothelial nitric oxide synthase to facilitate the release of nitric oxide and vasodilation.^21^, ^22^ Considering the association between vascular stiffness and metabolic inflexibility, similar to the ageing process,^23^ we hypothesised that Terazosin may also have efficacy on anti‐vascular stiffness. Therefore, we conducted a pilot study to investigate the effect of low‐dose of Terazosin on the improvement of arterial stiffness.

## 2 MATERIALS AND METHODS

### 2.1 Study design

The primary objective of this pilot study was to investigate the association between the administration of Terazosin and improvement in arterial stiffness among middle‐aged and older participants at Tongji Hospital from December 2020 to June 2023. The study protocol was approved by the Central Ethics Committee of Tongji Hospital, and all participants provided signed informed consent. The study protocol was registered at ClinicalTrials.gov (NO. ChiCTR2000040605).

We enrolled participants with elevated arterial stiffness (baPWV ≥1400 cm/s) and initiated low‐dose Terazosin (0.5 and 1.0 mg/day) upon completion of vascular stiffness assessment. Participants were followed at approximately 3‐month intervals for the Terazosin group. Exclusion criteria included age under 40 or over 85 years, pre‐existing cardiovascular diseases, cancer, pregnancy, hepatic or renal dysfunction, acute infection, etc. Additionally, individuals with abnormal glucose or lipid levels requiring hypoglycemic or lipid‐lowering medications, those receiving antihypertensive treatment, and individuals unable to comply with the prescribed treatment and follow‐up procedures were also excluded to mitigate confounding effects from non‐Terazosin arterial stiffness‐lowering drugs.

**FIGURE 1:**
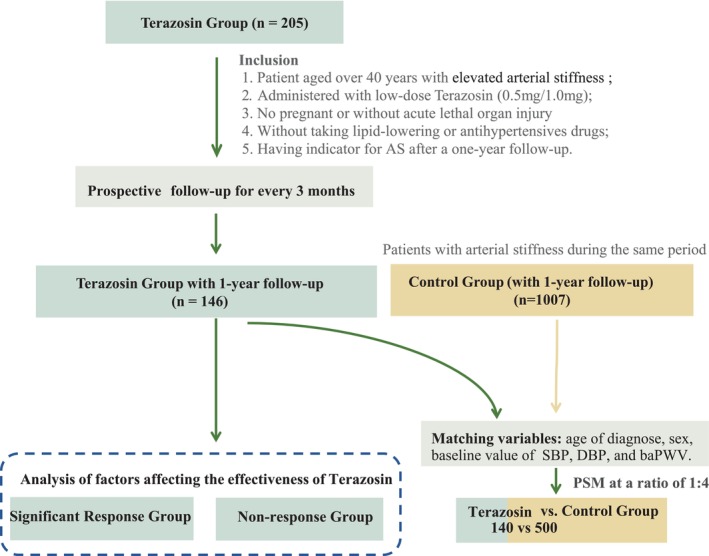
The flowchart showing the strategy of participant enrolment and subgroup analysis. Patients receiving Terazosin treatment were divided into two groups based on their response: the response group (showing at least a 5% reduction in baPWV) and the non‐response group (defined otherwise). Cases completed over 1 year of follow‐up were selected and further matched with Terazosin‐free controls. BaPWV, Brachial‐ankle pulse wave velocity; BMI, body mass index; DBP, diastolic blood pressure; MAP, Mean blood pressure; SBP, systolic blood pressure.

To characterise the improvement in baPWV associated with 1‐year Terazosin intake, cases in the Terazosin group who completed over 1 year of follow‐up were selected and further matched with Terazosin‐free controls. Considering that arterial stiffness is a significant risk factor for adverse events, it is imperative to advise patients on effectively managing abnormal blood pressure, blood glucose, blood lipid levels, and unhealthy lifestyle habits. To ensure maximum benefit for patients, it is crucial to avoid including forcibly assigned patients in an uncontrolled control group. Therefore, to minimise potential confounding factors, we retrospectively recruited patients diagnosed with arterial stiffness in our centre during the same period as the control group. The flowchart is presented in Figure 1.

### 2.2 Data collection

We proactively engage with patients in the Terazosin group every 3 months through telephone calls or text messages to provide reminders for their scheduled check‐ups. The control data was obtained from our centre's vascular health monitoring platform. In cases where patients exhibit signs of vascular stiffness during the examination, the platform will recommend an expansion of parameters for detecting baPWV and blood pressure in their subsequent annual examinations.

### 2.3 Assessment of arterial stiffness

BaPWV was measured using the Omron Colin BP‐203RPE III device (Omron Health Care, Kyoto, Japan). Four pneumatic pressure cuffs were attached at the bilateral brachia and ankles and connected to plethysmographic and oscillometric pressure sensors. Participants were instructed to rest in the supine position for at least 5 minutes under fasting conditions. The mean value of baPWV from the right and left sides was calculated. The response to treatment was determined by the formula, i.e., [(baPWV at treatment − baPWV at baseline)/baPWV at baseline] × 100. Individuals were divided into two groups according to their baseline arterial stiffness. Considering the balanced distribution of patients and the association between vascular stiffness and ageing, which often exhibits partial remission, we defined baPWV below 1600 as an improvement in vascular stiffness. Therefore, patients with baPWV ranging from 1400 cm/s to less than 1600 cm/s were classified as having moderate arterial stiffness, while those with baPWV equal to or exceeding 1600 cm/s were classified as having severe arterial stiffness.

### 2.4 Covariates

Age at vascular assessment was determined by calculating the interval between the date of birth and the baseline vascular assessment initiation date. BMI was calculated as weight (kg) divided by the square of height (m^2^). Overweight was defined as 24 kg/m^2^ ≤ BMI < 28 kg/m^2^, obesity was defined as BMI ≥28 kg/m^2^. Smoking status was classified as current smokers (those who smoked at least one pack per month for six consecutive months) and non‐smokers. Alcohol consumption was categorised as current drinkers (consuming alcohol at least once per week for six consecutive months) and non‐drinkers. Mean blood pressure (MAP) in mmHg was calculated as DBP+(SBP‐DBP)/3. Hypertension was defined according to JNC‐7 criteria here, which includes SBP ≥140 mmHg, DBP ≥90 mmHg, use of antihypertensive medication, or self‐reported history of hypertension.^24^ The diagnosis of dyslipidemia encompassed one or more of the following criteria, i.e., total cholesterol (TC) > 6.2 mmol/L, triglycerides (TG) ≥ 2.3 mmol/L, low‐density lipoprotein cholesterol (LDL‐C) ≥ 4.1 mmol/L, high‐density lipoprotein cholesterol (HDL‐C) ≤ 1.0 mmol/L, or under lipid‐lowering treatment according to the 2016 Chinese guidelines.^25^ The diagnosis of diabetes referred to fasting plasma glucose (FPG) ≥ 7.0 mmol/L, HbA1c ≥ 6.5%, the use of any antidiabetic medication, or self‐reported diagnosis history of diabetes.^26^

### 2.5 Statistical analysis

Continuous variables were described as mean ± standard deviation (SD) for normal distributions or median with interquartile range for skewed distributions, respectively, and examined by the Student's *t*‐test or ANOVA for normally distributed variables and Mann–Whitney U test or Kruskal–Wallis test for non‐parametric ones. Categorical variables were described as numbers (proportions) and examined by Fisher's exact test or χ^2^ test, as appropriate. Patients with missing information at baseline were excluded from the analysis, while for subsequent follow‐up data handling. The primary outcome was non‐response to the Terazosin, defined as a reduction in baPWV of less than 5% compared to the baseline. The follow‐up time was calculated from baseline to the end of the study or lost to follow‐up, whichever came first. The incidence rate of Terazosin effectiveness was calculated by dividing the number of incident cases by the total number observed during the follow‐up period. We performed logistic regression to test the risk factor for non‐response outcomes after adjusting for potential confounders, including age, sex, baseline baPWV, SBP, DBP, BMI, smoking, and alcohol consumption.

Cases who had been followed up over 1 year were further selected and matched with medication‐free controls for age, sex, age, SBP, DBP, and baPWV value at baseline by PSM at a ratio of 1:4. Then, linear regression analysis was performed to calculate the beta coefficients and 95% confidence intervals to represent the change in baPWV associated with Terazosin administration.

All statistical analyses were performed using R (version 4.0). Two‐sided *p* < 0.05 was considered statistically significant. Sensitivity analyses were performed to test the robustness of the results. The significant response of Terazosin intake was alternatively defined as a reduction in baPWV exceeding 10%.

## 3 RESULTS

### 3.1 Baseline characteristics of participants with Terazosin treatment

A total of 205 individuals with arterial stiffness who adhered to a regular Terazosin medication regimen were included. Of them, 137 (66.8%) were male, and 88 (42.9%) had hypertension (Table 1). Patients with severe arterial stiffness exhibited advanced age, higher baseline SBP, and a greater prevalence of hypertension compared to those with moderate arterial stiffness (Table 1).

**TABLE 1:** Baseline characteristics of the 205 patients Terazosin cohort stratified based on arterial stiffness.

Parameters	Total (*N* = 205)	Moderate arterial stiffness (*N* = 80)	Severe arterial stiffness (*N* = 125)	*p*‐value
Demographics				
Male, *n* (%)	137 (66.8%)	55 (68.8%)	82 (65.6%)	0.753
Age, year	57.1 ± 8.4	52.3 ± 6.7	60.2 ± 7.9	**<0.001**
BMI, kg/m^2^	24.9 ± 2.9	24.8 ± 3.3	25.1 ± 2.7	0.811
Normal weight, *n* (%)	78 (38.1%)	34 (42.5%)	44 (35.2%)	0.525
Overweight, *n* (%)	102 (49.8%)	36 (45.0%)	66 (52.8%)	
Obesity, *n* (%)	25 (12.2%)	10 (12.5%)	15 (12.0%)	
Current smokers, *n* (%)	48 (23.41%)	18 (22.5%)	30 (24.0%)	0.938
Current drinker, *n* (%)	63 (30.73%)	23 (28.8%)	40 (32.0%)	0.736
Medical conditions				
Hypertension, *n* (%)	88 (42.9%)	23 (28.8%)	65 (52.0%)	**0.002**
Diabetes, *n* (%)	31 (15.1%)	9 (11.3%)	22 (17.6%)	0.299
Dyslipidemia, *n* (%)	86 (42.0%)	33 (41.3%)	53 (42.4%)	0.986
Dose of Terazosin				
0.5 mg/day	77 (37.1%)	45 (56.3%)	62 (49.6%)	0.432
1.0 mg/day	98 (47.8%)	35 (43.8%)	63 (50.4%)	
Vascular variables				
Baseline baPWV, cm/s	1720.0 ± 259.3	1500.0 ± 55.6	1860.0 ± 238.0	**<0.001**
Brachial SBP, mm Hg	137.8 ± 15.7	132.0 ± 13.7	141.0 ± 15.9	**<0.001**
Brachial DBP, mm Hg	82.9 ± 10.3	81.3 ± 8.7	84.0 ± 11.1	0.073
Brachial MAP, mmHg	111.2 ± 20.2	109.0 ± 19.6	113.0 ± 20.5	0.098
Heart rate, bpm	69.9 ± 9.1	68.9 ± 8.4	70.5 ± 9.4	0.259
Laboratory variables				
Triglycerides, mg/dL	1.81 ± 1.35	1.92 ± 1.69	1.75 ± 1.03	0.934
TC, mmol/L	4.60 ± 1.04	4.61 ± 0.97	4.64 ± 1.08	0.746
LDL‐C, mg/dL	2.76 ± 0.89	2.78 ± 0.84	2.79 ± 0.94	0.934
HDL‐C, mg/dL	1.25 ± 0.52	1.25 ± 0.54	1.24 ± 0.49	0.935
FPG, mmol/L	5.76 ± 1.37	5.68 ± 1.76	5.81 ± 1.07	0.063

*Note*: Moderate arterial stiffness: 1400 cm/s < baPWV <1600 cm/s; Severe arterial stiffness: baPWV ≥1600 cm/s. Normal weight was defined as BMI < 24 kg/m^2^, Overweight was defined as 24 kg/m^2^ ≤ BMI < 28 kg/m^2^ and obesity was defined as BMI ≥28 kg/m^2^. All values are presented as *n* (%) and mean ± standard deviation. Independent sample *t*‐test for continuous variables and chi‐square test for categorical variables. Bold values indicate statistically significant *p*‐values (*p* < 0.05). Abbreviations: BaPWV, Brachial‐ankle pulse wave velocity; BMI, body mass index; DBP, diastolic blood pressure; FPG, fasting plasma glucose; HDL‐C, high‐density lipoprotein cholesterol; LDL‐C, low‐density lipoprotein cholesterol; MAP, mean blood pressure; SBP, systolic blood pressure; TC, total cholesterol.

### 3.2 Utilisation of Terazosin was associated with a significant improvement in arterial stiffness

**FIGURE 2:**
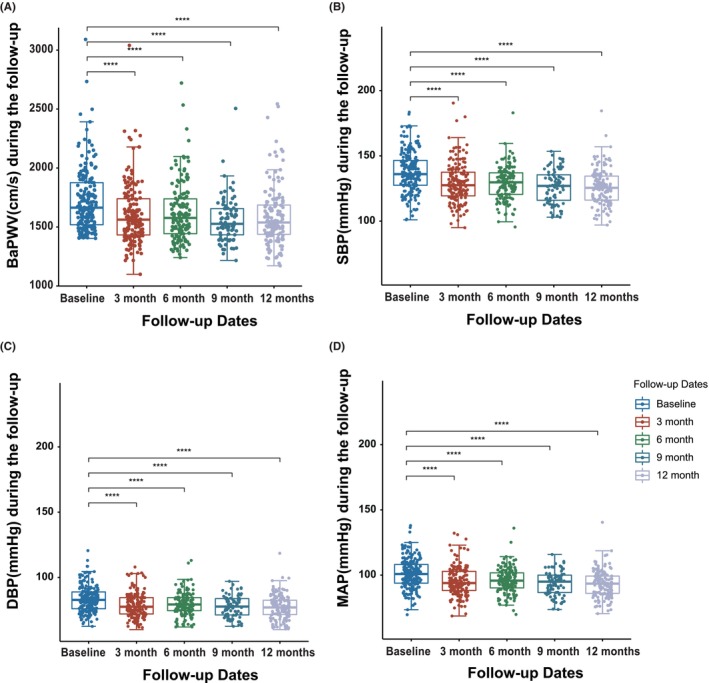
Longitudinal follow‐up of the Terazosin cohort every 3 months. (A) The mean ± SD of the baPWV response from baseline to 1‐year follow‐up was presented for the Terazosin group; (B‐D) The mean ± SD of the accompanying changes of SBP, DBP, and MAP were shown. BaPWV, brachial‐ankle pulse wave velocity; BMI, body mass index; DBP, diastolic blood pressure; MAP, mean blood pressure; SBP, systolic blood pressure; SD, standard deviation. ****P< 0.0001 compared to Baseline.

The longitudinal follow‐up revealed that the overall response rates of Terazosin from baseline to 3, 6, 9, and 12 months were 84/168 (50.0%), 71/146 (48.6%), 48/81 (59.3%), 87/146 (54.4%), respectively (Table 2). The baPWV value decreased from 1720.0 ± 259.3 cm/s at baseline to 1611.7 ± 263.8 cm/s after 3 months (*p* < 0.001) and 1592.7 ± 248.3 cm/s after 1 year (*p* < 0.001) of Terazosin administration, accompanied by a slight decline in blood pressure with consistent trends (Table 2; Figure 2).

**TABLE 2:** Treatments effects on vascular stiffness and incidence rate of significant efficacy after administration of Terazosin for per 3 months.

Parameters	Baseline	3 months	6 months	9 months	12 months
	*N* = 205	*N* = 168	*N* = 146	*N* = 81	*N* = 146
BaPWV, cm/s (*p* value)	1720.0 ± 259.3	1611.7 ± 263.8	1620.1 ± 246.9	1560.2 ± 193.3	1592.7 ± 248.3
		(*p* < 0.001)	(*p* < 0.001)	(*p* < 0.001)	(*p* < 0.001)
Brachial SBP, mm Hg (*p* value)	137.6 ± 15.5	129.1 ± 15.1	129.1 ± 12.6	126.6 ± 12.8	126.2 ± 13.5
		(*p* < 0.001)	(*p* < 0.001)	(*p* < 0.001)	(*p* < 0.001)
Brachial DBP, mm Hg (*p* value)	82.9 ± 10.3	78.4 ± 9.6	79.27 ± 9.2	77.3 ± 8.5	77.0 ± 9.5
		(*p* < 0.001)	(*p* < 0.001)	(*p* < 0.001)	(*p* < 0.001)
Brachial MAP, mmHg (*p* value)	101.1 ± 11.3	95.3 ± 10.9	95.9 ± 9.6	93.7 ± 9.4	93.4 ± 10.1
		(*p* < 0.001)	(*p* < 0.001)	(*p* < 0.001)	(*p* < 0.001)
Significant improvement in baPWV (≥ 5% reduction)		84 (50.0%)	71 (48.6%)	48 (59.3%)	87 (54.4%)

*Note*: Data are presented as mean ± SD or proportions. A paired *t*‐test was used for continuous variables conducted between the follow‐up and baseline periods. Abbreviations: BaPWV, Brachial‐ankle pulse wave velocity; BMI, body mass index; DBP, diastolic blood pressure; MAP, Mean blood pressure; SBP, systolic blood pressure; SD, standard deviation.

In the PSM cohort, 140 patients from the Terazosin group and 500 matched controls were randomly selected from the patient pool (Figure 1). The baseline characteristics were comparable between the two groups (Table 3). After 1 year of follow‐up, cases in the Terazosin group showed a lower mean of baPWV compared to Terazosin‐free controls (1580.9 vs. 1750.0 cm/s, *p* < 0.001) (Table S1; Figure S1A). This reduction was consistent across mean SBP (126.0 vs. 138.0 mmHg, *p* < 0.001), DBP (77.1 vs. 83.5 mmHg, *p* < 0.001), and MAP (93.3 vs. 102.0 mmHg, *p* < 0.001) (Table S1; Figure S1B–D). The administration of Terazosin at doses of 0.5 mg or 1.0 mg/day all has the potential to mitigate vascular stiffness (Figure S3A). Despite variations in the observed effects among groups, a consistent and significant reduction in BaPWV was demonstrated across all subgroups based on risk factors, including different age groups, baseline levels of vascular stiffness, and presence of hypertension (Figure S3B–D).

**TABLE 3:** Clinical characteristics of vascular stiffness patients in Terazosin group and control group before and after PSM at a ratio of 1:4.

Parameters	Unmatched			Matched		
	Control (*n* = 1007)	Terazosin (*n* = 146)	*p* Value	Control (*n* = 500)	Terazosin (*n* = 140)	*p* Value
Demographics						
Male, *n* (%)	714 (70.9%)	106 (72.6%)	0.745	357 (71.4%)	101 (72.1%)	0.947
Age, year	59.1 ± 10.8	57.5 ± 8.3	0.441	57.9 ± 10.9	57.4 ± 8.0	0.576
BMI, kg/m^2^	25.0 ± 3.0	24.9 ± 2.7	0.955	25.1 ± 3.2	25.0 ± 2.7	0.756
Normal weight, *n* (%)	390 (38.7%)	52 (35.6%)	0.337	192 (38.4%)	49 (35.0%)	0.268
Overweight, *n* (%)	469 (46.6%)	77 (52.7%)		228 (45.6%)	74 (52.9%)	
Obesity, *n* (%)	148 (14.7%)	17 (11.6%)		80 (16.0%)	17 (12.1%)	
Current drinker, *n* (%)	360 (35.7%)	47 (32.2%)	0.454	184 (36.8%)	45 (32.1%)	0.359
Current smokers, *n* (%)	326 (32.4%)	39 (26.7%)	0.201	76 (15.2%)	39 (27.9%)	**<0.001**
Medical conditions						
Hypertension, *n* (%)	414 (41.1%)	69 (47.3%)	0.188	220 (44.0%)	65 (46.4%)	0.678
Diabetes, *n* (%)	137 (13.6%)	23 (15.8%)	0.566	67 (13.4%)	21 (15.0%)	0.729
Dyslipidemia, *n* (%)	413 (41.0%)	63 (43.2%)	0.282	195 (39.0%)	62 (44.3%)	0.303
Vascular variables						
Baseline baPWV, cm/s	1641.3 ± 240.0	1725.6 ± 265.3	**<0.001**	1680.7 ± 262.4	1704.2 ± 247.1	0.327
Brachial SBP, mm Hg	134.0 ± 15.0	138.0 ± 15.7	**0.002**	136.0 ± 15.4	137.0 ± 15.2	0.376
Brachial DBP, mm Hg	83.6 ± 10.7	83.2 ± 10.1	0.456	83.0 ± 10.9	83.2 ± 9.9	0.793
Brachial MAP, mmHg	100.0 ± 10.9	102.0 ± 11.3	0.226	101.0 ± 11.1	101.0 ± 11.1	0.477
Heart rate, bpm	71.8 ± 11.3	69.6 ± 8.8	**0.016**	71.6 ± 11.8	69.8 ± 8.8	0.069

*Note*: Normal weight was defined as BMI < 24 kg/m^2^, Overweight was defined as 24 kg/m^2^ ≤ BMI < 28 kg/m^2^ and obesity was defined as BMI ≥28 kg/m^2^. *p* values for the group wise differences between Terazosin and controls were calculated by T test for normally distributed continuous variables and Fisher's exact test or χ^2^ test for categorical variables. Bold values indicate statistically significant *p*‐values (*p* < 0.05). Abbreviations: BaPWV, Brachial‐ankle pulse wave velocity; BMI, body mass index; DBP, diastolic blood pressure; MAP, mean blood pressure; PSM, propensity score matching; SBP, systolic blood pressure; SD, standard deviation.

Linear regression analysis revealed a statistically significant reduction in baPWV after 1 year of Terazosin administration, with an adjusted coefficient of −165.16 (95%CI: −224.85 to −105.46, *p* < 0.001). After stratifying the population based on age groups, vascular stiffness, and presence of hypertension, we observed a significant improvement in baPWV with Terazosin administration for both hypertensive and normotensive patients. However, individuals with hypertension showed more significant changes in on‐therapy baPWV. Similarly, more significant improvements were observed in individuals aged over 55 years or those with a baseline baPWV greater than 1600 cm/s (Table S2).

### 3.3 Univariate and multivariate analysis of risk factors for Terazosin response

We focused on 146 patients from the Terazosin group who underwent multiple follow‐up detection over a 1‐year period to identify the primary risk factors affecting the efficacy of Terazosin. Individuals were categorised according to their responses. The two patient groups exhibit variations in the baseline. Patients with good responses demonstrate a higher prevalence of hypertension, accompanied by elevated SBP and MAP levels (Table S3). Utilising a logistic model, individuals with higher baPWV than 1600 cm/s at baseline had a significantly lower risk of non‐response (OR: 0.337; 95% CI: 0.147–0.773, *p* = 0.010) (Table S4). Similarly, those with hypertension had a significantly lower risk of Terazosin non‐response than normotensive patients (OR: 0.486; 95% CI: 0.234–1.008, *p* = 0.053). No significant association was found in Terazosin dose between the two groups (*p* = 0.723). The 3‐month follow‐up revealed consistent results, suggesting a significantly reduced risk of non‐response to Terazosin in patients with higher baseline baPWV and elevated blood pressure for both the short‐ and long‐term (Tables S5 and S6). Notably, patients who did not respond well within the first 3 months had a significantly higher risk (OR = 3.313, 95% CI: 1.417–7.742, *p* = 0.006) of treatment non‐response at 12 months (Table S4).

### 3.4 Joint association of hypertension and arterial stiffness stratification with risk of non‐response to Terazosin

Considering the potential combined effects of multiple factors in clinical applications, a joint analysis of cumulative effects was done and showed that individuals diagnosed with hypertension and severe stiffness exhibited the lowest risk of non‐response to treatment compared to those with normal blood pressure and moderate arterial stiffness (OR = 0.220, 95% CI: 0.073–0.669, *p* = 0.007) (Table S7; Figure S2A). Additionally, patients below the age of 55 present with severe arterial stiffness had a lower risk of non‐response (OR = 0.059, 95% CI: 0.007–0.496, *p* = 0.009) (Table S8; Figure S2B). Sensitivity analyses produced consistent results (Table S9).

## 4 DISCUSSION

The results of longitudinal follow‐up in the Terazosin group indicate arterial stiffness improvement following treatment with Terazosin. In the PSM cohort, Terazosin treatment exhibited a significant impact on baPWV, with an annual adjusted reduction of 165 cm/s, irrespective of patient age or hypertension status. Furthermore, our findings suggest that patients with higher baseline baPWV or elevated blood pressure may derive greater benefits from Terazosin treatment.

Vascular stiffness is associated with adverse clinical outcomes, including stroke,^27^ myocardial infarction,^28^ heart failure,^29^ and premature death^30^ regardless of conventional cardiovascular disease risk factors including blood pressure.^31^ Therefore, improving vascular stiffness has become an important choice for preventing cardiovascular diseases. The process of ageing is a significant factor that contributes to the stiffening of major elastic arteries, accompanied by histological and biochemical alterations in artery walls.^32^, ^33^ Despite the progressive increase in collagen deposition, decrease in elastin quantities, and occurrence of vascular calcification observed during ageing,^33^, ^34^ the specific targets and pathways underlying anti‐arterial stiffness remain incompletely elucidated.

Research has demonstrated that certain antihypertensive drugs, such as angiotensin receptor blockers (ARBs), angiotensin‐converting enzyme inhibitors (ACE inhibitors), aldosterone antagonists, statins, calcium channel blockers, SGLT2 inhibitors, and metformin possess potential vascular benefits beyond their primary function of lowering blood pressure. Multiple studies consistently demonstrate the favourable impact of ARBs on arterial stiffness. For example, valsartan decreases arterial stiffness in hypertensive individuals independently of blood pressure reduction.^35^, ^36^ In a single‐blind trial, prolonged administration of irbesartan significantly decreases aortic stiffness and wave reflection in over 70% of hypertensive participants after 6 months.^37^ Furthermore, telmisartan effectively reduces arterial stiffness among hypertensive patients with Type 2 diabetes mellitus (T2DM),^38^ while olmesartan improves endothelial function and reduces vascular inflammation independently of its antihypertensive effects in individuals with uncontrolled essential hypertension.^39^, ^40^ Similarly, ACE inhibitors have demonstrated the ability to enhance arterial properties in hypertension patients. A study on perindopril found a correlation between blood pressure reduction and PWV during the first 2 months of treatment.^41^ Notably, after 6 months, PWV continued to decrease in the presence of stable blood pressure control, indicating significant arterial wall remodelling that remains unaffected by fluctuations in blood pressure.^41^ Similar effects of independent reductions in large artery stiffness were observed with quinapril and ramipril.^42^ Furthermore, aldosterone antagonists such as eplerenone have been reported to improve arterial elasticity and reduce arterial stiffness, associated with a decreased collagen/elastin ratio, improved arterial flexibility, and a reduction in circulating inflammatory mediators.^43^ Other classes of antihypertensive drugs have also shown potential in improving arterial stiffness. The use of fluvastatin, a statin medication, has effectively improved arterial stiffness in individuals with coronary artery disease and hyperlipidemia.^44^ This improvement correlates with reductions in LDL‐C and CRP levels and has been observed consistently over periods ranging from 12 months to 5 years.^44^ In individuals aged 60–75 years with newly diagnosed hypertension, the calcium channel blocker amlodipine effectively reduced intima‐media thickness. After 1 year of treatment, it demonstrated comparable improvements to the ACE inhibitor lisinopril,^45^ albeit a slight increase in intima‐media thickness was observed after the second year despite blood pressure control.^46^ Tofogliflozin, a selective inhibitor of SGLT2, has been reported to significantly reduce the cardio‐ankle vascular index in patients with T2DM after 6 months of starting treatment.^45^ Moreover, this therapeutic intervention elicits favourable alterations in anthropometric parameters including waist circumference, body weight, body mass index, as well as subcutaneous and visceral fat volume.^45^ Metformin has shown efficacy in improving arterial stiffness, blood pressure, and vascular responses in patients with polycystic ovary syndrome (PCOS), alongside reductions in weight, waist circumference, triglyceride levels, and increased adiponectin levels.^47^ Despite the aforementioned findings, current research predominantly focuses on the antihypertensive effects of these drugs, inadvertently revealing their potential to enhance vascular stiffness rather than directly addressing therapeutic interventions for mitigating vascular stiffening. Nonetheless, despite its potential to improve vascular stiffness, RAS blockade's impact on cardiovascular mortality remains suboptimal.^48^, ^49^ Therefore, there remains an imminent necessity to investigate novel pharmacological interventions targeting vascular stiffness.

Terazosin, an α1 adrenergic receptor antagonist traditionally used for treating benign prostatic hyperplasia and occasional hypertension at higher dosages (above 10 mg daily), has recently been discovered to enhance ATP generation by regulating glycolysis through binding to PGK1 and augmenting its kinase activity.^16^ This drug has shown potential in mitigating cellular pathophysiology associated with mitochondrial deficit phenotypes in Parkinson's disease.^17^ The increase in ATP levels has the potential to decelerate cellular ageing^20^ and augment the activity of endothelial nitric oxide synthase, leading to enhanced vasodilation.^21^, ^22^ These findings suggest that Terazosin could serve as a promising therapeutic approach for alleviating vascular stiffness due to its dual mechanism of regulating metabolic inflexibility and antagonizing the alpha‐1 adrenergic receptor. A preliminary study found that a daily dosage of 5 mg Terazosin significantly increased ATP levels in individuals with Parkinson's disease, without causing notable side effects or orthostatic hypotension. Higher doses did not provide additional benefits and showed a potential biphasic dose–response relationship.^50^ To minimise adverse effects from blood pressure reduction, we employed low doses of Terazosin (0.5 mg or 1.0 mg/day) to investigate its blood pressure‐independent positive impact on vascular stiffness in middle‐aged and elderly human subjects.

We compared the Terazosin study with previous reports on antihypertensive drugs. Previous research has shown that elevated arterial stiffness can precede the onset of high blood pressure, highlighting the importance of intervening in vascular stiffness even in non‐hypertensive patients.^31^ Our findings revealed that Terazosin significantly improved vascular stiffness in both groups, with consistent trends observed in blood pressure changes and arterial compliance improvements attributed to the medication.^51^, ^52^ No adverse effects from excessive blood pressure reduction were observed, indicating its safety and efficacy. Regression analysis results also showed that baPWV remained a significant predictor of drug effectiveness even after adjusting for blood pressure levels. Furthermore, through extensive documentation of patient responses following drug administration, we found that Terazosin exhibited its therapeutic effect 3 months after initiation and demonstrated sustained improvement throughout the 12‐month treatment period. Interestingly, it had a higher probability of continued effectiveness at 3 months. Additionally, we conducted subgroup comparisons based on baseline arterial stiffness levels and age to further analyse individual responses to Terazosin.

We found that individuals with baPWV ≥1600 cm/s had a significantly lower risk of non‐response, implying Terazosin might be particularly effective for individuals with severe arterial stiffness. Since hypertension is prevalent among the elderly due to age‐related degeneration of vascular elasticity and increased peripheral vascular resistance, it can be considered an inevitable aspect of ageing.^53^ Consistent with this, our findings indicate that patients with pre‐existing hypertension at baseline exhibited a reduced risk of Terazosin non‐response, potentially attributable susceptibility to vascular ageing, and impaired repair mechanisms in the context of poorly controlled hypertension.^54^ Therefore, it was reasonable that patients presenting both hypertension and severe arterial stiffness may potentially derive optimal benefits from Terazosin therapy in this study. Given the high prevalence of arterial stiffness among older adults, it is imperative to meticulously evaluate the efficacy of Terazosin treatment while considering patient age. We observed a more favourable response in individuals under 55 years old with severe baseline baPWV, suggesting potential significant vascular ageing and endothelial dysfunction within this specific subgroup. These findings provide valuable insights to inform the clinical selection of patients. It is worth noting that the clinical response to Terazosin treatment typically becomes evident after 3 months, with predictability for long‐term outcomes. No heterogeneity was observed in the risk for vascular improvement non‐response with varying doses of Terazosin usage, suggesting that lower dose (0.5 mg/day) Terazosin treatment remains effective. Analysis of associated risk factors provides potential research hints for exploring the mechanism of Terazosin.

Our study has several notable strengths. First, this is the first attempt to investigate the effect of regular low‐dose Terazosin administration on arterial stiffness improvement in a clinical trial population. Through longitudinal follow‐up, we described the temporal dynamics of patient response and observed a favourable response among the majority of patients. The detailed grouping analysis, conducted through univariate and joint analysis, yielded valuable research insights for investigating the primary risk factors associated with drug non‐response. Second, baPWV was employed as an assessment method for arterial stiffness in this study, not solely as a surrogate for carotid‐femoral pulse wave velocity (cfPWV); instead, it represents a distinct measure influenced by the properties of both the aorta and the muscular arteries in the lower extremities.^55^ Despite the significant implications of this study, it is essential to acknowledge its limitations. One notable limitation is the absence of randomised controls and a placebo effect in assessing treatment efficacy, which may introduce potential bias. However, it is important to note that this pilot study was designed as an exploratory investigation, and efforts were made to retrospectively collect control data and utilise PSM to minimise confounding factors arising from baseline differences among patients. Another limitation is the small‐scale and single‐centre nature of the study, conducted exclusively at Tongji Hospital. To further strengthen the reliability and generalizability of our findings, future research should involve multi‐centre studies with larger sample sizes. The subsequent randomised controlled trial (RCT) studies will include direct comparisons with traditional antihypertensive medications to enhance the clinical significance of the research. Additionally, it is worth highlighting the meticulous documentation and precision of our vascular ageing data platform, which significantly bolstered the rigour and validity of our results. These measures ensure that the findings are reliable and can be considered for potential translational applications. While these limitations should be taken into consideration, this study provides invaluable insights into the impact of low‐dose Terazosin on arterial stiffness. Despite its exploratory observational nature, this study holds significant translational relevance and offers valuable directions for future research in this field.

## 5 CONCLUSION

In summary, this pilot study provides valuable insights into the potential clinical significance of low‐dose Terazosin in improving arterial stiffness. Notably, these results pave the way for future randomised clinical trials, which have the potential to elucidate the full therapeutic potential of Terazosin in combating vascular ageing. If confirmed, the use of Terazosin could represent a major advancement in the prevention and treatment of cardiovascular diseases associated with arterial stiffness. Overall, the promising outcomes of this study underscore the urgent need for further research in this area and highlight the potential translational impact of our findings on patient care and disease management.

## AUTHOR CONTRIBUTIONS

**Yuqi Guan:** Conceptualization (equal); data curation (equal); formal analysis (equal); investigation (equal); methodology (lead); project administration (equal); software (lead); supervision (equal); visualization (lead); writing – original draft (lead); writing – review and editing (lead). **Yucong Zhang:** Data curation (equal); resources (equal); writing – review and editing (equal). **Liangkai Chen:** Data curation (equal); methodology (equal); software (equal); writing – review and editing (equal). **Yazhi Ren:** Data curation (equal); software (equal). **Hao Nie:** Data curation (equal); software (equal). **Tianyi Ji:** Data curation (equal); software (equal). **Jinhua Yan:** Funding acquisition (equal); writing – review and editing (equal). **Cuntai Zhang:** Funding acquisition (lead); writing – review and editing (equal). **Lei Ruan:** Supervision (equal); validation (equal); writing – review and editing (equal).

## FUNDING INFORMATION

This work was supported by the National Natural Science Foundation of China (Grant Numbers: 82101635; principal investigator: J.Y) and the National Key Research and Development Program of China (Grant Number: 2020YFC2008000; principal investigator: C.Z).

## CONFLICT OF INTEREST STATEMENT

The authors declare that the research was conducted in the absence of any commercial or financial relationships that could be construed as a potential conflict of interest.

## Supporting information


Figure S1.

Figure S2.

Figure S3.

Table S1.
Table S2.
Table S3.
Table S3.
Table S4.
Table S5.
Table S6.
Table S7.
Table S8.
Table S9.

## Data Availability

Data available in article supplementary material .
