# Supplementary figures and images for: Effect of low‐dose terazosin on arterial stiffness improvement: A pilot study
Source: J Cell Mol Med. 2024 Jul 23;28(14):e18547. doi: 10.1111/jcmm.18547 (PMC11265993; doi:10.1111/jcmm.18547)

**A**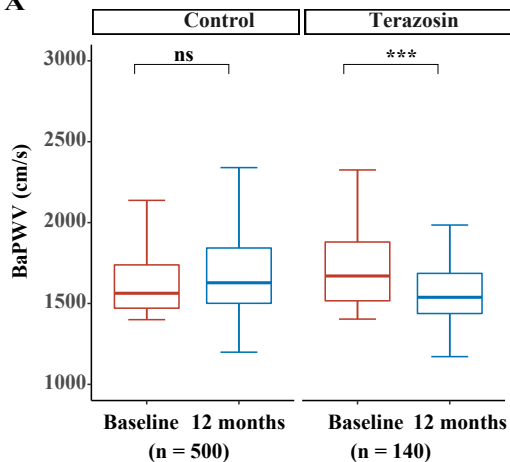**B**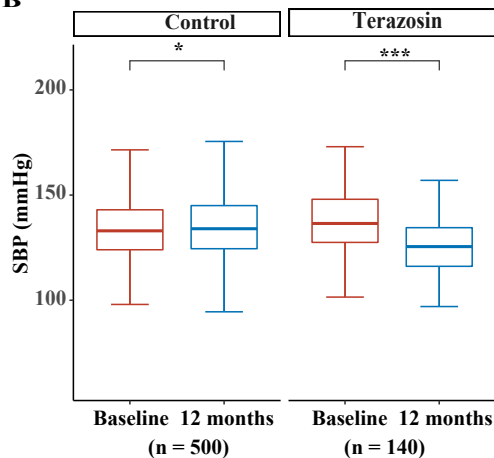**C**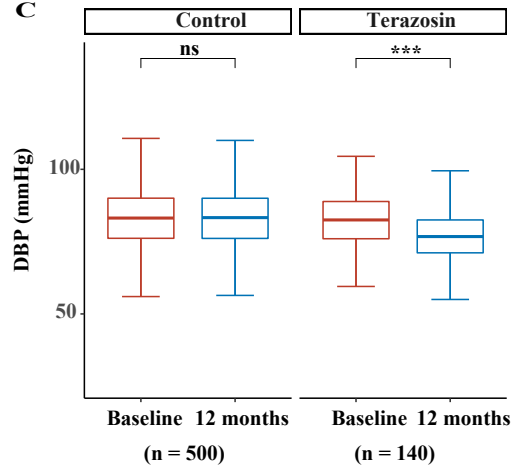**D**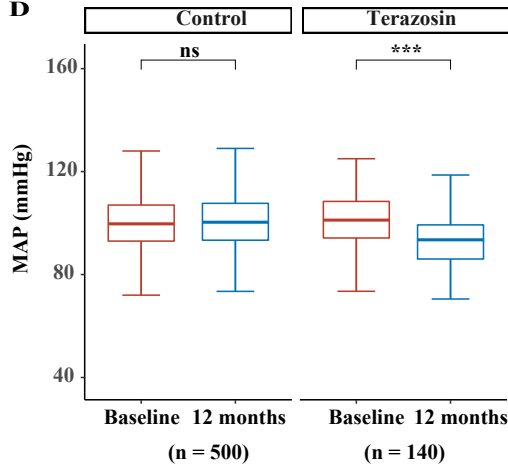

Follow-up Dates

Baseline  
12 months

Supplement: Supplementary file 1 — Figure S1. [file JCMM-28-e18547-s003.pdf]

**A.**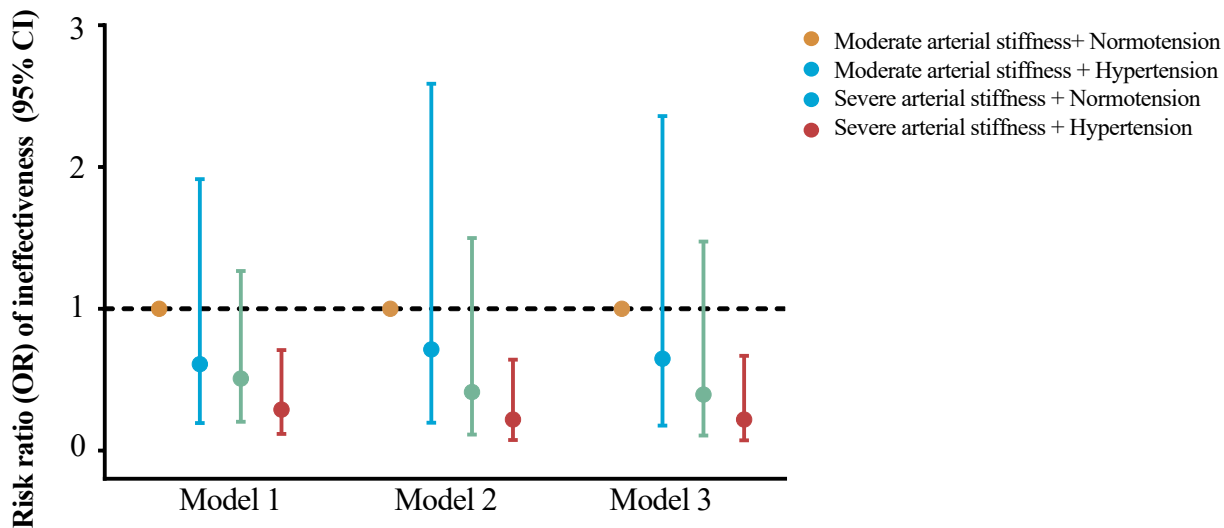**B.**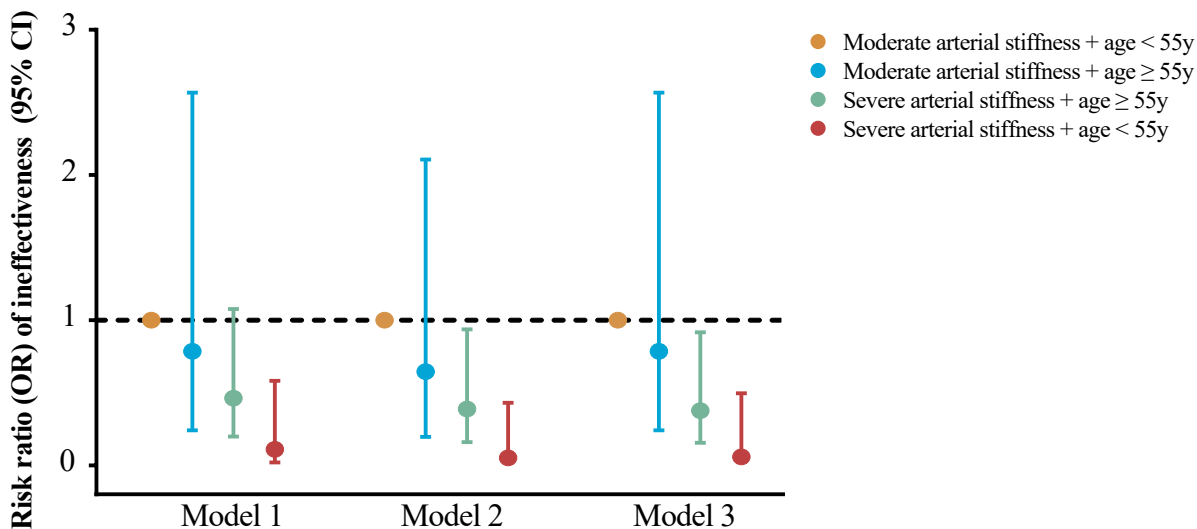

Supplement: Supplementary file 2 — Figure S2. [file JCMM-28-e18547-s004.pdf]

A

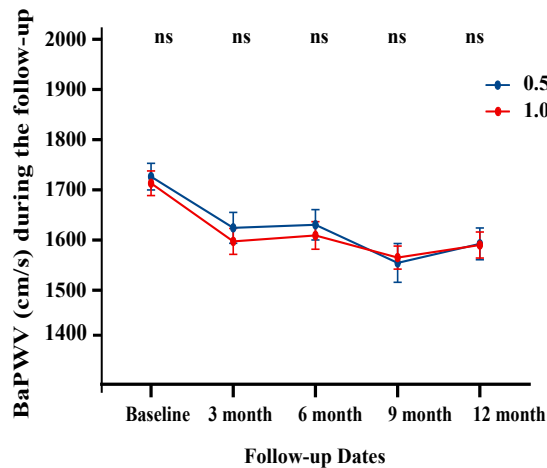

B

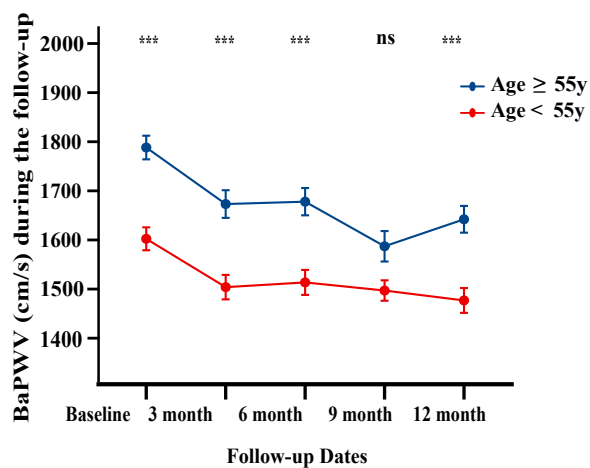

C

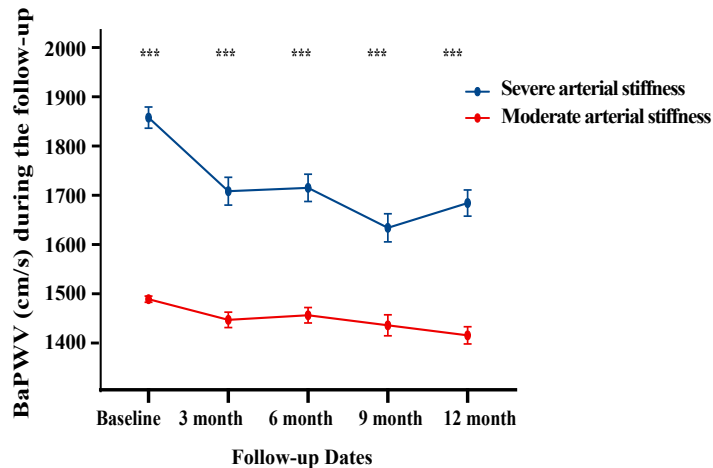

D

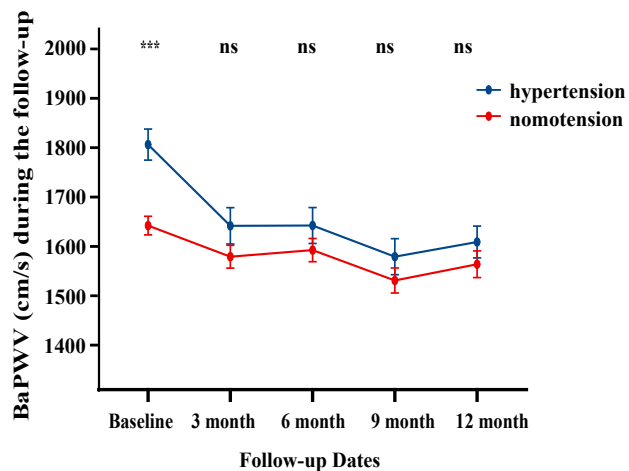

Supplement: Supplementary file 3 — Figure S3. [file JCMM-28-e18547-s002.pdf]
