# Supplementary material for: Effect of low‐dose terazosin on arterial stiffness improvement: A pilot study
Source: J Cell Mol Med. 2024 Jul 23;28(14):e18547. doi: 10.1111/jcmm.18547 (PMC11265993; doi:10.1111/jcmm.18547)
Supplement: Supplementary file 4 — Table S1. Table S2. Table S3. Table S3. Table S4. Table S5. Table S6. Table S7. Table S8. Table S9. [file JCMM-28-e18547-s001.docx]

**Supplementary Material**

**Supplemental methods**

**Figure S1．**Improved arterial stiffness after one-year administration of Terazosin in the PSM cohort.

**Figure S2.** Joint impact of hypertension or advanced age within arterial stiffness stratification on the risk of non-response to Terazosin.

**Figure S3.** Subgroup analysis for longitudinal follow-up of the Terazosin cohort.

**Table S1.** The improvement on the vascular stiffness associated with one-year Terazosin treatment in PSM cohort.

**Table S2.** Subgroup analyses for the associations between the on-treat in baPWV increase with Teazosin intake for 12 months in PSM cohort.

**Table S3.** Clinical baseline characteristics of 146 cases in the Terazosin group who were followed up over 12 months.

**Table S3.** The improvement in arterial stiffness improvement in the Terazosin group after one-year treatment (n = 146).

**Table S4.** Risk factors for non-response outcomes in 146 patients following 12-months administration of Terazosin.

**Table S5.** Baseline characteristics of Terazosin group stratified by 3-month drug response (n=168).

**Table S6.** Factors associated with on-treat baPWV after 3-month Terazosin administration (n=168).

**Table S7.** Factors analysis associated with the risk of non-response to Terazosin by the arterial stiffness and hypertension status.

**Table S8.** The impact of baseline baPWV and age group on the risk of non-response to Terazosin.

**Table S9.** Sensitivity analyses for associations between clinical bassline factors with risk of non-response outcomes after 12-month Terazosin.

**Figure S1. Improved arterial stiffness after one-year administration of Terazosin in the PSM cohort.**


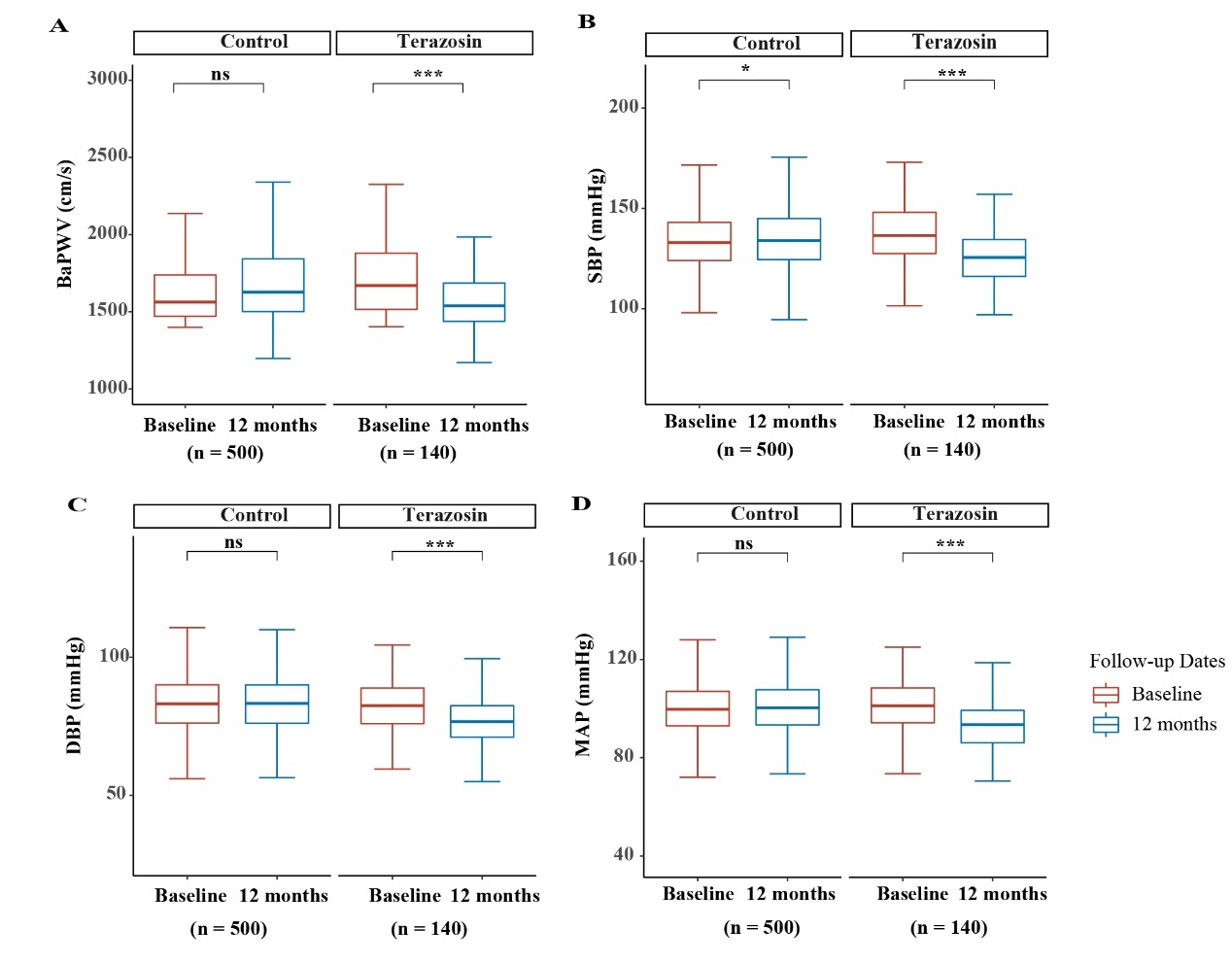


**Note.** SBP, systolic blood pressure; DBP, diastolic blood pressure; MAP, Mean blood pressure; BMI, body mass index; BaPWV, Brachial-ankle pulse wave velocity; PSM, propensity score matching; Ns, P > 0.05; * P ≤ 0.05; ** P ≤ 0.01; *** P ≤ 0.001.

**Figure Caption:** (A) The baseline and 12-month baPWV values were compared between 140 Tearzosin case and 500 matched control cases post PSM;(B-D) Comparison of SBP, DBP and MAP after PSM analysis.

**Figure S2.** **Joint associations of hypertension and arterial stiffness with the risk of Terazosin's non-response.**


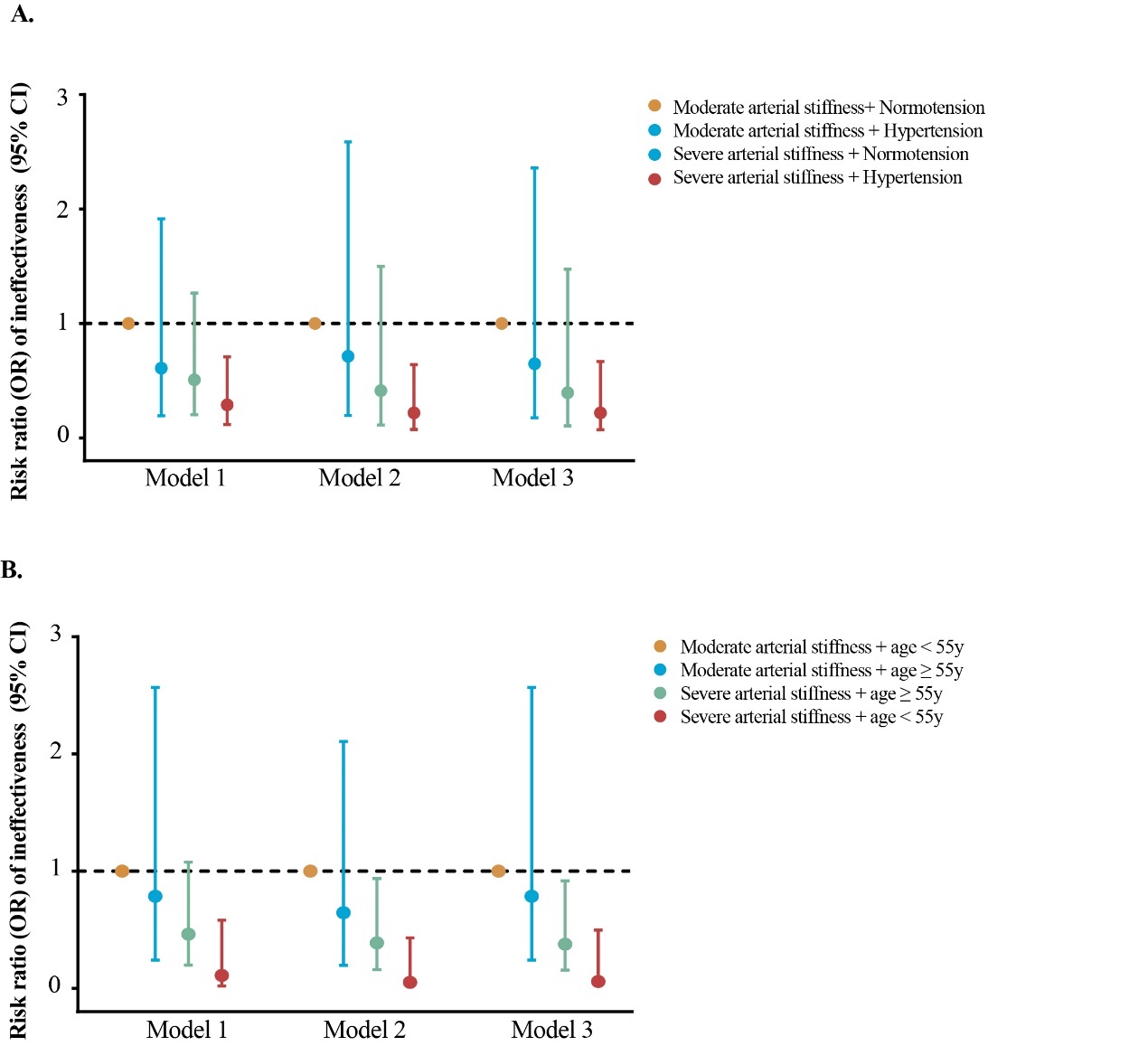


Note. OR, odd ratio; CI, confidence interval; SBP, systolic blood pressure; DBP, diastolic blood pressure; MAP, Mean blood pressure; BMI, body mass index; BaPWV: Brachial-ankle pulse wave velocity; Model 1: Crude model; Model 2 was adjusted for age (continuous), sex, smoking, alcohol and BMI; Model 3 was adjusted for age (continuous), sex, smoke, alcohol, BMI, diabetes and dyslipidemia. Moderate arterial stiffness, 1400 cm/s < baPWV < 1600 cm/s; Severe arterial stiffness: baPWV ≥1600 cm/s.

Figure Caption: (A). The risk of Terazosin's non-response significantly reduced in young patients with severe arterial stiffness. (B). Patients aged less than 55 with severe arterial stiffness exhibited a significant decrease in non-response to Terazosin when analyzing age subgroups and arterial stiffness jointly.

**Figure S3. Subgroup analysis for longitudinal follow-up of the Terazosin cohort.**

**
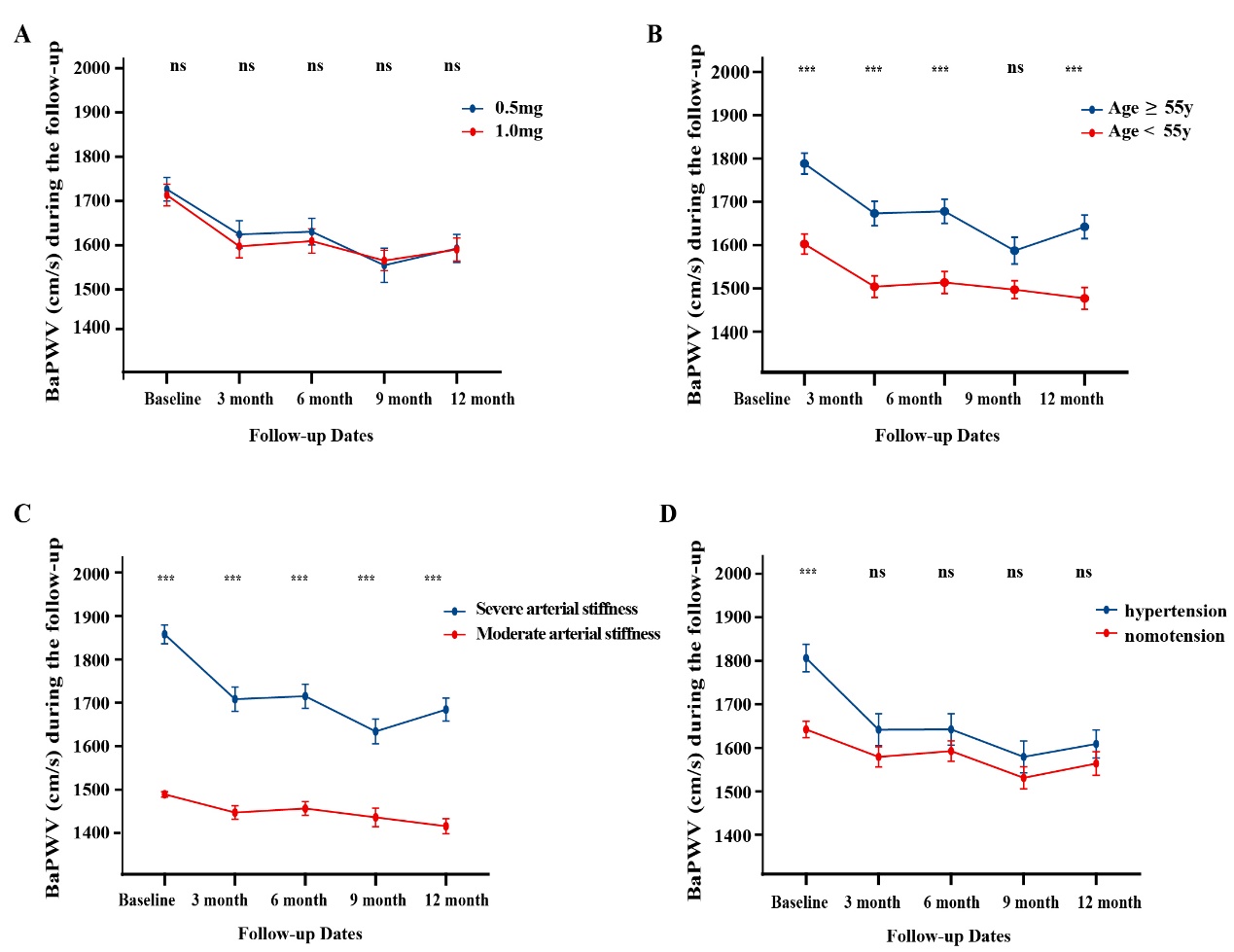
**

Note. BaPWV: Brachial-ankle pulse wave velocity; Moderate arterial stiffness, 1400 cm/s < baPWV < 1600 cm/s; Severe arterial stiffness: baPWV ≥1600 cm/s.

Figure Caption: The mean ± SD of the baPWV response from baseline to 1-year follow-up was evaluated in individuals categorized based on Terazosin doses (0.5 mg or 1.0 mg/day) (A), as well as age, baseline baPWV, and hypertension status (B-D).

##### Table S1. The improvement on the vascular stiffness associated with one-year Terazosin treatment in PSM cohort.

| **Parameters** | **Control** | **Terazosin** | ***P*-value** |
| --- | --- | --- | --- |
|  | **(N=500)** | **(N=140)** |  |
| BMI, kg/m^2^ | 25.0 ± 3.1 | 24.6 ± 2.7 | 0.104 |
| 1-year baPWV, cm/s | 1750.0 ± 334.8 | 1580.9 ± 248.9 | < 0.001 |
| Brachial SBP, mm Hg | 138.0 ± 16.7 | 126.0 ± 13.5 | < 0.001 |
| Brachial DBP, mm Hg | 83.5 ± 11.3 | 77.1 ± 9.50 | < 0.001 |
| Brachial MAP, mm Hg | 102.0 ± 11.7 | 93.3 ± 10.2 | < 0.001 |
| Heart rate, bpm | 72.2 ± 12.1 | 69.6 ± 9.05 | 0.006 |

**Note.** PSM, propensity score matching; BMI, body mass index; BaPWV: Brachial-ankle pulse wave velocity; SBP, systolic blood pressure; DBP, diastolic blood pressure; MAP, Mean blood pressure.

##### Table S2. Subgroup analyses for the associations between the on-treat in baPWV increase with Teazosin intake for 12 months in PSM cohort.

| **Subgroup** | **Model 1** | | **Model 2** | | **Model 3** | |
| --- | --- | --- | --- | --- | --- | --- |
|  | ***STβ***  **(95% CI)** | **P value** | ***STβ***  **(95% CI)** | **P value** | ***STβ***  **(95% CI)** | **P value** |
| **Age** |  |  |  |  |  |  |
| ＜ 55 years | -126.34  (-187.21--65.48) | < 0.001 | -120.89  (-181.96--59.824) | < 0.001 | -125.91  (-187.30--64.52) | < 0.001 |
| ≥ 55 years | -224.89  (-305.62--144.17) | < 0.001 | -142.86  (-212.49--73.22) | < 0.001 | -145.47  (-215.03--75.91) | < 0.001 |
| **Baseline baPWV** |  |  |  |  |  |  |
| < 1600 cm/s | -105.45  (-143.44--67.45) | < 0.001 | -101.07  (-139.07--63.07) | < 0.001 | -102.32  (-140.44--64.20) | < 0.001 |
| ≥ 1600 cm/s | -276.53  (-355.50--197.57) | < 0.001 | -229.66  (-298.95--160.36) | < 0.001 | -230.93  (-300.01--161.84) | < 0.001 |
| **Hypertension** |  |  |  |  |  |  |
| no | -112.71  (-181.80--43.61) | 0.001 | -127.89  (-185.66--70.12) | < 0.001 | -127.96  (-185.94--69.97) | < 0.001 |
| yes | -234.65  (-332.38--136.93) | < 0.001 | -183.12  (-264.26--101.98) | < 0.001 | -174.26  (-255.17--93.34) | < 0.001 |

**Note.** SBP, systolic blood pressure; DBP, diastolic blood pressure; MAP, Mean blood pressure; BMI, body mass index; BaPWV: Brachial-ankle pulse wave velocity; CI, confidence interval. Standardized regression coefficients (ST*β*) indicate the association of Terazosin intake with baPWV Change. All analyses were adjusted using Model 1: Crude model, Model 2: age (continuous), sex, smoking, alcohol and BMI and Model 3: age (continuous), sex, smoke, alcohol, BMI, diabetes and dyslipidemia, excluding factors associated with the analyzed variables.

##### Table S3. Clinical baseline characteristics of 146 cases in the Terazosin group who were followed up over 12 months.

| **Parameters** | **Non-response Group** | **Response Group** | ***P* value** |
| --- | --- | --- | --- |
|  | **(N=59)** | **(N=87)** |  |
| **Demographics** |  |  |  |
| Male, n (%) | 38 (64.4%) | 68 (78.2%) | 0.101 |
| Age, year | 57.0 ± 7.9 | 57.8 ± 8.5 | 0.566 |
| Current drinking (n, %) | 18 (30.5%) | 29 (33.3%) | 0.859 |
| Current smoking (n, %) | 18 (30.5%) | 21 (24.1%) | 0.507 |
| BMI, kg/m^2^ | 24.4 (3.03) | 25.3 (2.31) | 0.035 |
| Normal weight, n (%) | 29 (49.2%) | 23 (26.4%) | 0.013 |
| Overweight, n (%) | 23 (39.0%) | 54 (62.1%) |  |
| Obesity, n (%) | 7 (11.9%) | 10 (11.5%) |  |
| **Medical conditions** |  |  |  |
| Diabetes, n (%) | 11 (18.6%) | 12 (13.8%) | 0.577 |
| Dyslipidemia, n (%) | 26 (44.1%) | 37 (42.5%) | 0.989 |
| Hypertension, n (%) | 22 (37.3%) | 47 (54.0%) | 0.069 |
| Dose of Terazosin |  |  |  |
| 0.5mg | 33 (55.9%) | 43 (49.4%) | 0.546 |
| 1.0mg | 26 (44.1%) | 44 (50.6%) |  |
| **Vascular variables** |  |  |  |
| Baseline BaPWV, cm/s | 1645.5 ± 214.2 | 1780.0 ± 283.4 | **0.001** |
| Moderate arterial stiffness, n (%) | 29 (49.2%) | 26 (29.9%) | **0.029** |
| Severe arterial stiffness, n (%) | 30 (50.8%) | 61 (70.1%) |  |
| Brachial SBP, mm Hg | 133.7 ± 13.8 | 141.2 ± 16.3 | **0.003** |
| Brachial DBP, mm Hg | 80.5 ± 8.6 | 85.1 ± 10.7 | **0.004** |
| Brachial MAP, mmHg | 98.2 ± 9.8 | 104.0 ± 11.8 | **0.002** |
| Heart rate, bpm | 70.7 ± 8.4 | 68.8 ± 9.0 | 0.189 |
| **Laboratory variables** |  |  |  |
| Triglycerides, mg/dL | 1.64 ± 0.81 | 1.96 ± 1.70 | 0.136 |
| TC, mmol/L | 4.68 ± 1.06 | 4.55 ± 0.95 | 0.456 |
| LDL-C, mg/dL | 2.90 ± 0.93 | 2.68 ± 0.84 | 0.160 |
| HDL-C, mg/dL | 1.21 ± 0.29 | 1.21 ± 0.54 | 0.923 |
| FPG mmol/L | 5.75 ± 0.96 | 5.79 ± 1.72 | 0.860 |

**Note.** SBP: systolic blood pressure; DBP: diastolic blood pressure; MAP: Mean blood pressure; BMI: body mass index; BaPWV: Brachial-ankle pulse wave velocity; TC: total cholesterol; HDL-C: high-density lipoprotein cholesterol; LDL-C: low-density lipoprotein cholesterol; FPG: fasting plasma glucose. The response group was defined as individuals who showed a more than 5 % absolute improvement in baPWV compared to the baseline, while the non-response group included not meet this criterion. Moderate arterial stiffness, 1400 cm/s < baPWV < 1600 cm/s; Severe arterial stiffness: baPWV ≥1600 cm/s. Normal weight was defined as BMI < 24 kg/m^2^, Overweight was defined as 24 kg/m^2^ ≤ BMI < 28 kg/m^2^ and obesity was defined as BMI ≥ 28 kg/m^2^. Independent sample *t*-test for continuous variables and chi-square test for categorical variables.

##### Table S4. Risk factors for non-response outcomes in 146 patients following 12-months administration of Terazosin.

| **Variables** | **Model 1** | | **Model 2** | | **Model 3** | |
| --- | --- | --- | --- | --- | --- | --- |
|  | **OR** | ***P*** | **OR** | ***P*** | **OR** | ***P*** |
|  | **(95% CI)** | **Value** | **(95% CI)** | **Value** | **(95% CI)** | **Value** |
| **Demographics** |  |  |  |  |  |  |
| Age, year | 0.988 (0.950-1.028) | 0.556 | - | - | - | - |
| Male, n (%) | 0.506 (0.241-1.059) | 0.071 | - | - | - | - |
| Current smoking, n (%) | 1.380 (0.656-2.901) | 0.396 | - | - | - | - |
| Current drinking , n (%) | 0.621 (0.420-0.919) | 0.017 | - | - | - | - |
| BMI, kg/m^2^ | 0.861 (0.735-1.009) | 0.065 | - | - | - | - |
| Normal weight, n (%) | Ref. | - | - | - | - | - |
| Overweight, n (%) | 0.338 (0.162-0.705) | 0.004 | - | - | - | - |
| Obesity, n (%) | 0.555 (0.182-1.691) | 0.3 | - | - | - | - |
| **Medical conditions** |  |  |  |  |  |  |
| Diabetes, n (%) | 1.432 (0.584-3.515) | 0.433 | 1.540 (0.627-3.785) | 0.347 | - | - |
| Dyslipidemia, n (%) | 0.640 (0.445-0.921) | **0.016** | 1.248 (0.605-2.575) | 0.549 | - | - |
| Hypertension, n (%) | 0.506 (0.257-0.997) | **0.049** | 0.502 (0.246-1.025) | **0.058** | 0.486 (0.234-1.008) | **0.053** |
| Dose of Terazosin |  |  |  |  |  |  |
| 1.0mg/day | Ref. | - | Ref. | - | Ref. | - |
| 0.5mg/day | 1.299 (0.667-2.528) | 0.442 | 1.157 (0.561-2.387) | 0.692 | 1.140 (0.551-2.358) | 0.723 |
| **Vascular variables** |  |  |  |  |  |  |
| Baseline BaPWV, cm/s | 0.998 (0.996-0.999) | **0.006** | 0.997 (0.995-0.999) | **0.004** | 0.997 (0.995-0.999) | **0.003** |
| Moderate arterial stiffness, n (%) | Ref. | - | Ref. | - | Ref. | - |
| Severe arterial stiffness, n (%) | 0.441 (0.221-0.878) | **0.020** | 0.345 (0.151-0.787) | **0.011** | 0.337 (0.147-0.773) | **0.010** |
| Brachial SBP, mm Hg | 0.967 (0.946-0.989) | **0.003** | 0.965 (0.942-0.990) | **0.006** | 0.964 (0.940-0.990) | **0.007** |
| Brachial DBP, mm Hg | 0.951 (0.918-0.986) | **0.006** | 0.952 (0.914-0.992) | **0.018** | 0.950 (0.910-0.992) | **0.020** |
| Brachial MAP, mmHg | 0.952 (0.922-0.984) | **0.003** | 0.952 (0.917-0.988) | **0.009** | 0.950 (0.913-0.988) | **0.011** |
| Non-response at 3 month | 3.325 (1.508-7.333) | **0.003** | 3.738 (1.640-8.522) | **0.002** | 3.313 (1.417-7.742) | **0.006** |

**Note.** OR, odd ratio; CI, confidence interval; SBP, systolic blood pressure; DBP, diastolic blood pressure; MAP, Mean blood pressure; BMI, body mass index; BaPWV: Brachial-ankle pulse wave velocity; Ref.: Reference. The primary outcome (non-response to Terazosin) was characterized by an absolute improvement in baPWV of less than 5 percent compared to the baseline. All variables were measured at the time of the study. Moderate arterial stiffness, 1400 cm/s < baPWV < 1600 cm/s; Severe arterial stiffness: baPWV ≥1600 cm/s. Model 1: Crude model; Model 2 was adjusted for age (continuous), sex, smoking, alcohol and BMI; Model 3 was adjusted for age (continuous), sex, smoke, alcohol, BMI, diabetes and dyslipidemia.

##### Table S5. Baseline characteristics of Terazosin group stratified by 3-month drug response (n=168).

| **Parameters** | **Non-response Group** | **Response**  **Group** | ***P* value** |
| --- | --- | --- | --- |
|  | **(N=84)** | **(N=84)** |  |
| **Demographics** |  |  |  |
| Male, n (%) | 54 (64.3%) | 58 (69.0%) | 0.623 |
| Age, year | 56.0 ± 8.2 | 57.7 ± 8.0 | 0.186 |
| Current drinking (n, %) | 27 (32.1%) | 25 (29.8%) | 0.867 |
| Current smoking (n, %) | 22 (26.2%) | 17 (20.2%) | 0.465 |
| BMI, kg/m^2^ | 24.9 ± 2.8 | 24.8 ± 3.0 | 0.735 |
| Normal weight, n (%) | 32 (38.1%) | 34 (40.5%) | 0.868 |
| Overweight, n (%) | 42 (50.0%) | 42 (50.0%) |  |
| Obesity, n (%) | 10 (11.9%) | 8 (9.5%) |  |
| **Medical conditions** |  |  |  |
| Diabetes, n (%) | 19 (22.6%) | 9 (10.7%) | 0.062 |
| Dyslipidemia, n (%) | 35 (41.7%) | 31 (36.9%) | 0.636 |
| Hypertension, n (%) | 28 (33.3%) | 44 (52.4%) | **0.019** |
| Dose of Terazosin |  |  |  |
| 0.5mg/day | 43 (51.2%) | 47 (56.0%) | 0.643 |
| 1.0mg/day | 41 (48.8%) | 37 (44.0%) |  |
| **Vascular variables** |  |  |  |
| Baseline BaPWV, cm/s | 1644.9 ± 237.7 | 1799.2 ± 275.4 | **< 0.001** |
| Moderate arterial stiffness, n (%) | 43 (51.2%) | 24 (28.6%) | **0.005** |
| Severe arterial stiffness, n (%) | 41 (48.8%) | 60 (71.4%) |  |
| Brachial SBP, mm Hg | 134.0 ± 13.3 | 141.0 ± 16.7 | **0.007** |
| Brachial DBP, mm Hg | 81.8 ± 9.25 | 84.4 ± 11.2 | 0.107 |
| Brachial MAP, mmHg | 110.2 ± 19.5 | 112.3 ± 20.4 | 0.507 |
| Heart rate, bpm | 70.3 ± 9.47 | 70.3 ± 9.61 | 0.983 |
| **Laboratory variables** |  |  |  |
| Triglycerides, mg/dL | 1.78 ± 1.15 | 1.68 ± 0.94 | 0.539 |
| TC, mmol/L | 4.65 ± 0.93 | 4.71 ± 1.04 | 0.711 |
| LDL-C, mg/dL | 2.84 ± 0.85 | 2.86 ± 0.86 | 0.915 |
| HDL-C, mg/dL | 1.25 ± 0.54 | 1.23 ± 0.30 | 0.822 |
| FPG mmol/L | 5.94 ± 1.86 | 5.60 ± 0.84 | 0.144 |

**Note.** SBP, systolic blood pressure; DBP, diastolic blood pressure; MAP, Mean blood pressure; BMI, body mass index; BaPWV: Brachial-ankle pulse wave; velocity; FPG: fasting plasma glucose. The response group was defined as individuals who showed more than 5 percent absolute reduction in baPWV compared to the baseline, while the non-response group included not meet this criterion.

##### Table S6. Factors associated with on-treat baPWV after 3-month Terazosin administration (n=168)

| **Variables** | **Model 1** | | **Model 2** | | **Model 3** | |
| --- | --- | --- | --- | --- | --- | --- |
|  | **OR**  **(95% CI)** | ***P***  **Value** | **OR**  **(95% CI)** | ***P***  **Value** | **OR**  **(95% CI)** | ***P***  **Value** |
| **Demographics** |  |  | . |  |  |  |
| Age, year | 0.975 (0.938-1.013) | 0.192 | . | . | . | . |
| Male, n (%) | 0.807 (0.423-1.537) | 0.514 | . | . | . | . |
| Current smoking, n (%) | 1.398 (0.679-2.882) | 0.363 | . | . | . | . |
| Current drinking , n (%) | 1.118 (0.580-2.155) | 0.739 | . | . | . | . |
| BMI, kg/m^2^ | 0.966 (0.864-1.081) | 0.546 | . | . | . | . |
| Normal weight, n (%) | Ref. | . | . | . | . | . |
| Overweight, n (%) | 1.062 (0.556-2.029) | 0.854 | . | . | . | . |
| Obesity, n (%) | 1.328 (0.464-3.798) | 0.597 | . | . | . | . |
| **Medical conditions** |  |  |  |  |  |  |
| Diabetes, n (%) | 2.777 (1.137-6.780) | **0.025** | 3.224 (1.308-7.947) | **0.011** | . | . |
| Dyslipidemia, n (%) | 1.221 (0.656-2.275) | 0.529 | 1.245 (0.637-2.431) | 0.521 | . | . |
| Hypertension, n (%) | 0.455 (0.243-0.850) | **0.014** | 0.411 (0.211-0.800) | **0.009** | 0.392 (0.193-0.797) | **0.010** |
| Dose of Terazosin |  |  |  |  |  |  |
| 1.0mg/day | Ref. | . | Ref. | . | Ref. | . |
| 0.5mg/day | 0.826 (0.449-1.518) | 0.537 | 0.880 (0.452-1.713) | 0.707 | 0.903 (0.456-1.785) | 0.769 |
| **Vascular variables** |  |  |  |  |  |  |
| Baseline BaPWV, cm/s | 0.997 (0.995-0.999) | **0.008** | 0.997 (0.995-0.999) | **0.012** | 0.997 (0.995-0.999) | **0.005** |
| Moderate arterial stiffness, n (%) | Ref. | . | Ref. | **.** | Ref. | **.** |
| Severe arterial stiffness, n (%) | 0.360 (0.189-0.685) | **0.002** | 0.333 (0.163-0.682) | **0.003** | 0.311 (0.149-0.65) | **0.002** |
| Brachial SBP, mm Hg | 0.972 (0.951-0.993) | **0.011** | 0.967 (0.946-0.989) | **0.003** | 0.966 (0.943-0.99) | **0.005** |
| Brachial DBP, mm Hg | 0.976 (0.947-1.005) | 0.107 | 0.969 (0.938-1.001) | 0.058 | 0.97 (0.937-1.005) | 0.090 |
| Brachial MAP, mmHg | 0.993 (0.979-1.008) | 0.371 | 0.991 (0.975-1.007) | 0.266 | 0.991 (0.975-1.007) | 0.266 |

**Note.** OR, odd ratio; CI, confidence interval; SBP, systolic blood pressure; DBP, diastolic blood pressure; MAP, Mean blood pressure; BMI, body mass index; BaPWV: Brachial-ankle pulse wave velocity; Ref.: Reference. All analyses were adjusted using Model 1: Crude model, Model 2: age (continuous), sex, smoking, alcohol and BMI and Model 3: age (continuous), sex, smoke, alcohol, BMI, diabetes and dyslipidemia, excluding factors associated with the analyzed variables.

##### Table S7. Factors analysis associated with the risk of non-response to Terazosin by the arterial stiffness and hypertension status

| **Joint** **factors** | **Incidence rate of non-response** | **Model 1** | | **Model 2** | | **Model 3** | |
| --- | --- | --- | --- | --- | --- | --- | --- |
|  |  | **OR**  **（95%CI）** | ***P* Value** | **OR**  **（95%CI）** | ***P* Value** | **OR**  **（95%CI）** | ***P* Value** |
| Moderate arterial stiffness+ Normotension | 56.8% (21/37) | Ref. | . | Ref. | . | Ref. | . |
| Moderate arterial stiffness + Hypertension | 44.4% (8/18) | 0.610  (0.194-1.916) | 0.397 | 0.713  (0.196-2.588) | 0.606 | 0.647  (0.177-2.360) | 0.510 |
| Severe arterial stiffness + Normotension | 40.0% (16/40) | 0.508  (0.204-1.266) | 0.146 | 0.412  (0.113-1.500) | 0.179 | 0.394  (0.106-1.474) | 0.167 |
| Severe arterial stiffness + Hypertension | 26.0% (13/50) | 0.288  (0.117-0.709) | **0.007** | 0.220  (0.075-0.641) | **0.006** | 0.220  (0.073-0.669) | **0.008** |

**Note.** CI, confidence interval; OR, odds ratio; Ref., reference. SBP, systolic blood pressure; DBP, diastolic blood pressure; MAP, Mean blood pressure; BMI, body mass index; BaPWV: Brachial-ankle pulse wave velocity. Model 1: Crude model, model. Model 2 was adjusted for age (continuous), sex, smoking, alcohol and BMI; Model 3 was adjusted for age (continuous), sex, smoke, alcohol, BMI, diabetes and dyslipidemia. Moderate arterial stiffness：1400 cm/s < BaPWV < 1600 cm/s; severe arterial stiffness: BaPWV ≥ 1600 cm/s.

##### Table S8. The impact of baseline baPWV and age group on the risk of non-response to Terazosin

| **Joint** **factors** | **Incidence rate of non-response** | **Model 1** | | **Model 2** | | **Model 3** | |
| --- | --- | --- | --- | --- | --- | --- | --- |
|  |  | **OR**  **（95%CI）** | ***P* Value** | **OR**  **（95%CI）** | ***P* Value** | **OR**  **（95%CI）** | ***P* Value** |
| Moderate arterial stiffness + age < 55y | 56.3% (18/32) | Ref. | . | Ref. | . | Ref. | . |
| Moderate arterial stiffness + age ≥ 55y | 47.8% (11/23) | 0.786  (0.241-2.567) | 0.690 | 0.645  (0.197-2.107) | 0.467 | 0.786  (0.241-2.567) | 0.690 |
| Severe arterial stiffness + age ≥ 55y | 37.3% (28/75) | 0.463  (0.199-1.078) | 0.074 | 0.389  (0.161-0.938) | **0.035** | 0.378  (0.156-0.917) | **0.031** |
| Severe arterial stiffness + age < 55y | 12.5% (2/16) | 0.111  (0.021-0.582) | **0.009** | 0.052  (0.006-0.431) | **0.006** | 0.059  (0.007-0.496) | **0.009** |

**Note.** CI, confidence interval; OR, odds ratio; Ref., reference. SBP, systolic blood pressure; DBP, diastolic blood pressure; MAP, Mean blood pressure; BMI, body mass index; BaPWV: Brachial-ankle pulse wave velocity. Adjusted for different models. Model 1: crude; Model 2: adjusted for age (continuous), sex, smoke, drink and BMI; Model 3: adjusted for age (continuous), sex, smoke, drink, BMI, diabetes and dyslipidemia.

##### Table S9. Sensitivity analyses for associations between clinical bassline factors with risk of non-response outcomes after 12-month Terazosin.

| **Risk Factors** | **Incidence rate of non-response** | **OR (95% CI)** | ***P* value** |
| --- | --- | --- | --- |
| **Multivariate analysis** | | | |
| Severe arterial stiffness , n (%) | - | 0.251 (0.099-0.637) | **0.004** |
| Baseline baPWV, cm/s | - | 0.997 (0.995-0.998) | **< 0.001** |
| Hypertension , n (%) | - | 0.524 (0.251-1.094) | 0.085 |
| Brachial SBP, mm Hg | - | 0.969 (0.943-0.996) | **0.026** |
| Brachial DBP, mm Hg | - | 0.961 (0.920-1.003) | 0.071 |
| Brachial MAP, mmHg | - | 0.959 (0.921-0.998) | **0.040** |
| Non-response in first 3 month | - | 6.216 (2.551-15.148) | **< 0.001** |
| **Subgroup Analysis 1** | | | |
| Normotension+ Moderate arterial stiffness | 75.67% (28/37) | Ref. | . |
| Normotension+ Severe arterial stiffness | 57.50% (23/40) | 0.435 (0.111-1.707) | 0.232 |
| Hypertension + Moderate arterial stiffness | 72.22% (13/18) | 1.187 (0.229-6.162) | 0.838 |
| Hypertension + Severe arterial stiffness | 42.00% (21/50) | 0.178 (0.056-0.566) | **0.003** |
| **Subgroup Analysis 2** | | | |
| Moderate arterial stiffness + age < 55y | 78.13% (25/32) | Ref. | . |
| Moderate arterial stiffness + age ≥ 55y | 69.57% (16/23) | 0.451 (0.107-1.903) | 0.278 |
| Severe arterial stiffness + age ≥ 55y | 50.67% (38/75) | 0.229 (0.082-0.644) | **0.005** |
| Severe arterial stiffness + age < 55y | 37.50% (6/16) | 0.169 (0.040-0.712) | **0.015** |

**Note.** OR, odd ratio; SBP, systolic blood pressure; DBP, diastolic blood pressure; MAP, Mean blood pressure; BMI, body mass index; BaPWV: Brachial-ankle pulse wave velocity; Moderate arterial stiffness, 1400 cm/s < baPWV < 1600 cm/s; Severe arterial stiffness: baPWV ≥1600 cm/s; Ref., reference. Sensitivity analysis: The significant response of Terazosin intake was alternatively defined as a reduction in baPWV exceeding 10%. All analyses were adjusted for age (continuous), sex, smoke, alcohol, BMI, diabetes and dyslipidemia, excluding factors associated with the analyzed variables.
